# Transparent reporting of multivariable prediction models for individual prognosis or diagnosis: checklist for systematic reviews and meta-analyses (TRIPOD-SRMA)

**DOI:** 10.1136/bmj-2022-073538

**Published:** 2023-05-03

**Authors:** Kym I E Snell, Brooke Levis, Johanna A A Damen, Paula Dhiman, Thomas P A Debray, Lotty Hooft, Johannes B Reitsma, Karel G M Moons, Gary S Collins, Richard D Riley

**Affiliations:** 1 Institute of Applied Health Research, College of Medical and Dental Sciences, University of Birmingham, Birmingham B15 2TT, UK; 2 Centre for Prognosis Research, School of Medicine, Keele University, Keele, UK; 3 Cochrane Netherlands, Julius Centre for Health Sciences and Primary Care, University Medical Centre Utrecht, Utrecht University, Utrecht, Netherlands; 4 Julius Centre for Health Sciences and Primary Care, University Medical Centre Utrecht, Utrecht University, Utrecht, Netherlands; 5 Centre for Statistics in Medicine, Nuffield Department of Orthopaedics, Rheumatology and Musculoskeletal Sciences, University of Oxford, Oxford, UK; 6 NIHR Oxford Biomedical Research Centre, Oxford University Hospitals NHS Foundation Trust, Oxford, UK

## Abstract

Most clinical specialties have a plethora of studies that develop or validate one or more prediction models, for example, to inform diagnosis or prognosis. Having many prediction model studies in a particular clinical field motivates the need for systematic reviews and meta-analyses, to evaluate and summarise the overall evidence available from prediction model studies, in particular about the predictive performance of existing models. Such reviews are fast emerging, and should be reported completely, transparently, and accurately. To help ensure this type of reporting, this article describes a new reporting guideline for systematic reviews and meta-analyses of prediction model research.

Clinical prediction models are used to predict health related outcomes in individual patients.^1^ ^2^ These models focus on predicting whether outcomes are either already present (diagnostic models) or will occur in the future (prognostic models).^3^ ^4^ Examples include the 4C Deterioration model for estimating risk of in-hospital clinical deterioration in adults with covid-19,^5^ the QRISK3 model for calculating risk of cardiovascular disease onset within 10 years,^6^ and the Wells’ score for estimating the risk of a deep vein thrombosis in individuals admitted to hospital with suspected deep vein thrombosis.^7^

Box 1 includes a glossary of terms relating to prediction models. Prediction model research includes primary studies of model development and model evaluation (validation).^8^ ^9^ Model development studies typically use statistical or machine learning methods to derive a model (eg, regression equation, random forest, or neural network) for predicting a specific outcome (eg, cardiovascular disease within 10 years) based on multiple variables (predictors) such as age, stage of disease, comorbidities, and biomarkers. Model validation studies evaluate an existing model’s predictive performance, for example, in terms of calibration, discrimination, overall fit, and clinical utility.^1^ ^2^ It is common to distinguish between internal and external validation.^10^ In an internal validation, performance is evaluated within the model development dataset itself, potentially while estimating and adjusting for optimism due to overfitting. Conversely, an external validation evaluates performance in data that were not used for model development,^9^ ^10^ ^11^ ^12^ ^13^ potentially even from a different target population or setting.^14^

Box 1 **Glossary of common terms used in prediction model and systematic review research**

**Prediction model**

A model (eg, based on a regression equation or a neural network) that predicts an outcome value (eg, blood pressure, weight) or outcome risk (eg, risk that a particular disease is present, or risk of a particular event occurring within 10 years) for an individual based on their values of multiple predictors. When the model predictions aim to inform diagnosis, it is a diagnostic model. When the model predictions aim to inform prognosis, it is a prognostic model.

**Report**

A document (paper or electronic) supplying information about a particular study. It could be a journal article, preprint, conference abstract, study register entry, clinical study report, dissertation, unpublished manuscript, government report, or any other document providing relevant information.

**Record**

The title or abstract (or both) of a report indexed in a database or website (such as a title or abstract for an article indexed in Medline). Records that refer to the same report (such as the same journal article) are duplicates; however, records that refer to reports that are merely similar (eg, a similar abstract submitted to two different conferences) should be considered unique.

**Study**

A research investigation that uses data from a defined group of participants to develop or validate a prediction model. A “study” might have multiple reports. For example, reports could include the protocol, statistical analysis plan, and a main article providing the model developed or estimates of model performance from a validation. Often a single study will include both model development and model validation.

**Model development**

The process of producing a model for predicting outcome values or calculating event risks in new individuals, typically undertaken using statistical or machine learning methods.

**Model validation**

The process of evaluating the predictive performance of a model; that is, checking whether the predictions from the model are accurate.

**Internal validation**

A validation of model performance that uses the same dataset as was used for model development, which includes deriving estimates of apparent performance (simply the observed model performance, without adjustment for optimism due to overfitting) and optimism adjusted estimates of performance (based on repeated resampling of the development dataset (eg, using bootstrapping or cross validation)).

**External validation**

An evaluation of model performance in a dataset different to that used for model development, often from a different population or setting.

**Model performance measures**

Statistics that quantify the accuracy of a model’s predictions, for example, in terms of calibration, discrimination, overall fit, and clinical utility.

**Calibration**

The agreement between predicted and observed outcome values, for example, as visualised using a calibration plot of observed versus predicted values (including a smoothed flexible calibration curve) and quantified by measures such as the calibration slope (ideal value is 1) and observed/expected value (ideal value is also 1).

**Discrimination**

How well a model’s risk predictions separate between those who have (diagnostic models) or develop (prognostic models) the outcome and those who do not have or do not develop the outcome. Discrimination is usually measured by the concordance (C) statistic (index), and a value of 1 indicates the model has perfect discrimination, while a value of 0.5 indicates the model discriminates no better than chance.

**Overall fit**

Measures summarising the difference between observed and predicted values; for example, R^2^ (the proportion of the total variance of outcome values that is explained by the model) or the mean squared error (also known as the Brier score for binary and survival outcomes).

**Clinical utility**

The overall benefit of using a model’s predictions to direct clinical decision making, for example, in terms of impact on patient and healthcare outcomes. Often measured by the net benefit, which weighs the benefits (eg, improved patient outcomes) against the harms (eg, worse patient outcomes).

**Systematic review**

A review that uses explicit, systematic methods to collate and synthesise findings of studies that answer a clearly formulated question.

**Meta-analysis**

A statistical technique used to synthesise estimates (eg, of model performance) from multiple studies, yielding a quantitative summary.

**PICOTS**

A system for framing the prediction model review question in terms of target population, index model(s), comparator model(s), outcome(s) to be predicted, timing (start point of prediction and time horizon for the prediction) and setting.

Glossary adapted from Page et al.^30^

Estimates of model performance might be imprecise from either internal or external validation, and single studies might not fully reflect the target population and setting for model deployment. Hence, multiple validation studies of the same prediction model are ideally conducted, each examining the performance of that model in a particular setting and population, and potentially comparing it against any other competing models. When multiple validation studies exist, this motivates the need for systematic reviews to identify, appraise, synthesise (meta-analyse), and summarise the evidence to support and compare prediction models in a particular field. For example, Lee et al present a systematic review of diagnostic models for paediatric foreign body aspiration,^15^ Kreuzberger et al provide a systematic review of prognostic models for outcomes in newly diagnosed chronic lymphocytic leukaemia in adults,^16^ and Damen et al use meta-analysis to summarise the performance of the Framingham model for prediction of cardiovascular disease risk at 10 years.^17^

Guidance for undertaking systematic reviews and meta-analyses of prediction model studies has been proposed by members of the Cochrane Prognosis Methods Group and other researchers.^2^ ^18^ ^19^ In general, researchers are recommended to define a PICOTS (population, index model(s), comparator model(s), outcome(s), timing, and setting) system for framing the research question^18^; search filters for identifying prediction model studies^20^ ^21^; and use the CHARMS (checklist for critical appraisal and data extraction for systematic reviews of prediction modelling studies) tool for extracting information from primary studies,^22^ PROBAST (prediction model risk-of-bias assessment tool) for evaluating the quality and applicability of prediction model studies,^23^ ^24^ statistical methods for obtaining estimates and confidence intervals of model performance measures from each study,^18^ ^25^ meta-analysis methods for combining and summarising study estimates of model performance,^18^ ^25^ and GRADE (grading of recommendations, assessment, development, and evaluations) proposals for evaluating the overall certainty of evidence.^26^

Complete, accurate, and transparent reporting is another essential part of a systematic review of prediction model studies. Reporting guidelines exist for primary studies that develop or validate a prediction model (TRIPOD (transparent reporting of multivariable prediction models for individual prognosis or diagnosis))^27^ ^28^ and for the reporting of systematic reviews of other research types, such as interventions (PRISMA (preferred reporting items for systematic reviews and meta-analyses^29^ ^30^)) or test accuracy (PRISMA-DTA^31^). However, no guidelines exist for reporting systematic reviews or meta-analyses of prediction model studies, which have unique challenges and issues beyond those covered by the existing TRIPOD and PRISMA guidelines. For example, in reviews examining the performance of a prediction model, measures of interest for meta-analysis include calibration and discrimination, whereas PRISMA focuses on intervention effects; and when examining applicability and quality (risk of bias), there needs to be clear differentiation between model development and model validation studies, which is not a concept in reviews of intervention studies. The need for specific reporting guidance was also recognised by the Cochrane Prognosis Methods Group in various reviews of prediction model studies over the past decade. Furthermore, the number of systematic reviews of this type will only increase with the rising interest in models developed using machine learning and artificial intelligence techniques.

Therefore, in this article, we propose a new guideline for the transparent reporting of multivariable prediction models for individual prognosis or diagnosis tailored for systematic reviews and meta-analyses (TRIPOD-SRMA). We describe the intended scope of TRIPOD-SRMA, describe the consensus process used to produce and finalise the items within TRIPOD-SRMA, and provide an overview of the checklist and how to use it.

**Summary points**

Clinical prediction models use a combination of variables to predict health outcomes in individuals, for example, to inform diagnosis or prognosis
For many healthcare domains and clinical fields, multiple competing prediction models exist, and multiple studies are available that examine and compare their predictive performance
Systematic reviews and meta-analyses of prediction model research identify, appraise, and summarise the evidence about existing models and their predictive performance, to help ascertain whether models are fit for purpose or to compare the performance between competing models
Systematic reviews should be completely, transparently, and accurately reported; to encourage this, TRIPOD-SRMA is a new reporting guideline for systematic reviews and meta-analyses of prediction model research
TRIPOD-SRMA contains 26 items and builds on previous reporting guidelines (most notably, PRISMA and TRIPOD); a corresponding TRIPOD-SRMA checklist for abstracts is also provided, containing 12 items

## Scope of TRIPOD-SRMA

TRIPOD-SRMA is a reporting guideline for any systematic review of prediction model studies, which might also include meta-analysis. Typically, such reviews have one or more of the following aims:

- To identify all prediction models within a particular clinical specialty
- To identify all prediction models for a particular target population
- To identify all prediction models for a particular outcome
- To summarise the predictive performance of one particular prediction model
- To summarise and compare the predictive performance of two or more prediction models.

By “prediction model” we mean a multivariable model that predicts an outcome value or risk for an individual person based on their values of multiple predictors (also known as variables, covariates, features, or characteristics, among others; box 1). Examples of typical published systematic reviews of prediction model studies that fall within the scope of TRIPOD-SRMA are provided in box 2. We emphasise that our focus is on reviews of models aiming for individualised outcome prediction. Hence, TRIPOD-SRMA is not intended for reviews that focus on the effect of particular factors or variables, such as in reviews examining the prognostic effect of a factor or meta-analyses summarising the interaction between a factor and treatment effect.^32^ ^33^ It also does not cover reviews of prediction model impact studies,^34^ ^35^ such as comparative studies (eg, randomised trials) evaluating the downstream consequences of using a prediction model in practice compared with not using a model, because these studies are more akin to intervention reviews.

Box 2 **Examples of different types of prediction model reviews covered by TRIPOD-SRMA**

TRIPOD-SRMA is a reporting guideline for any systematic review of prediction model studies (with or without meta-analysis). Examples of such reviews include:

- A systematic review to identify, synthesise, and compare existing clinical prediction models designed to support the diagnosis of asthma in children and adults presenting with symptoms suggestive of asthma in primary care or equivalent settings.^37^
- A living systematic review of covid-19 prediction models,^38^ aiming to identify and appraise the validity and usefulness of any models for the diagnosis of covid-19 in patients with suspected infection, prognosis of patients with covid-19, or identification of people in the general population at increased risk of covid-19 infection or being admitted to hospital with the disease.
- A systematic review of prediction models for cardiovascular disease risk in the general population,^39^ aiming to provide an overview of any studies describing the development or external validation of a prediction model for risk of incident cardiovascular disease.
- A systematic review and meta-analysis of any studies evaluating the performance of the EuroSCORE,^40^ a prediction model for the risk of operative mortality after cardiac surgery.
- A systematic review of studies examining the performance of the MELD (model for end stage liver disease) score for estimating the probability of survival after transplantation in adults receiving liver transplants,^41^ including a qualitative summary of study findings (without meta-analysis).
- A systematic review and meta-analysis of studies developing or validating prognostic models for complete recovery in ischemic stroke,^42^ aiming to summarise both discrimination and calibration performance of each model.
- A systematic review of prognostic models for newly diagnosed chronic lymphocytic leukaemia in adults, aiming to identify, describe, and appraise any models developed to predict overall survival, progression-free survival, or treatment-free survival, and including meta-analysis to summarise their predictive performances.
- A systematic review to evaluate the evidence on comparisons of established cardiovascular risk prediction models and to collect comparative information on their relative performance.^43^

TRIPOD-SRMA focuses on reviews that use aggregate information (eg, summaries of the characteristics of study participants, estimates, and confidence intervals of a model’s predictive performance) extracted from study publications or obtained from study authors. It is not intended to cover individual participant data meta-analysis,^19^ where the raw data are obtained and synthesised from each study, as that situation is covered by TRIPOD-Cluster.^36^

## Development of TRIPOD-SRMA

An executive committee was set up (comprising the authors of this article), and included members of the original TRIPOD collaboration (www.tripod-statement.org).^27^ ^28^ This committee then led the development of TRIPOD-SRMA, following guidance published by Moher et al^44^ and the associated EQUATOR Network toolkit for developing a reporting guideline (https://www.equator-network.org/). Although TRIPOD-SRMA borrows heavily on PRISMA and PRISMA 2020 (see below), after discussion among the executive committee (and agreement with the senior author of PRISMA 2020), we took the decision to label the checklist as a TRIPOD guideline to fit in the family of reporting guidelines that focuses solely on diagnostic and prognostic prediction models, rather than as an extension of the PRISMA family.

At the first project meeting in September 2019, the varied aims of systematic reviews of prediction model studies were discussed and the remit of the new reporting guidance was agreed. Existing reporting guidelines and other relevant documents were identified and included TRIPOD,^27^ TRIPOD-Cluster (which was simultaneously being developed by members of the executive group),^36^ PRISMA,^45^ PRISMA-DTA,^31^ PRISMA-S,^46^ the templates for Cochrane prognosis reviews (https://methods.cochrane.org/prognosis/tools), and critical appraisal and risk-of-bias tools including CHARMS and PROBAST.^22^ ^23^

Following the initial meeting, two investigators (KIES, BL) reviewed the identified documents and led the development of the initial draft TRIPOD-SRMA checklist based on items from the existing guidelines (in particular, TRIPOD, TRIPOD-Cluster, and PRISMA). The process involved identifying relevant items from the existing guidelines, modifying some of the items as considered necessary, and adding new items where needed. All items included in the draft checklist were discussed with the wider executive group, initially at an in-person meeting (February 2020) and then in more detail at a virtual meeting (June 2020), together with correspondence over email.

Once the executive committee agreed on the items that should be included in the checklist, a modified Delphi process was used to elicit the views from a wider group of experts through online surveys. Ethics approval to conduct the surveys was obtained from the ethics committee of Keele University’s Faculty of Medicine and Health Sciences Research. The aims of the Delphi surveys were to inform the consensus process on which reporting items to include or exclude from the checklist, and to gather opinions and feedback to refine the wording of included items. Researchers (statisticians, clinical epidemiologists, systematic reviewers, and clinicians) with expertise in primary studies or systematic reviews of (diagnostic or prognostic) prediction model studies were invited to participate in the first Delphi survey via email in June 2021. The survey remained open for four weeks, during which time two reminders were sent. Of 86 individuals invited, 43 participated in the survey, forming the Delphi panel. Thirty (70%) and 22 (51%) participants had experience in systematic reviews of prognostic and diagnostic models, respectively. Thirty six (84%) and 19 (44%) participants had experience of developing and validating prognostic and diagnostic models, respectively. Six individuals also mentioned other relevant experience, such as methodology research or systematic reviews of prognostic factor studies. The survey was conducted through Keele Health Survey, powered by LimeSurvey.^47^

In this first survey, the Delphi panel were asked to state how strongly they thought each item should be included in the checklist, using a five point Likert scale ranging from “strongly agree” to “strongly disagree.” Participants were also able to leave comments for each item. The executive committee then met virtually in September 2021 to discuss the Delphi results, in particular to identify items where consensus agreement was not obtained among the Delphi participants, while also considering free text suggestions and comments. Consensus agreement was defined a priori as having at least two thirds of participants agreeing with the item, consistent with other Delphi studies used to inform reporting guidelines and conducted by members of the executive committee. Therefore, items were updated and modified accordingly to produce a revised TRIPOD-SRMA checklist, which was agreed among the executive committee in November 2021. Items from existing reporting guidelines (in particular, PRISMA) were only modified where the feedback deemed it clearly necessary, in order to maintain consistency with items already familiar to systematic reviewers. While developing the checklist, PRISMA 2020 was published, superseding the original PRISMA checklist. Therefore, several items in the TRIPOD-SRMA checklist were also amended to reflect the updated items in PRISMA 2020.

Participants who had responded to the first survey were invited to participate in a second Delphi survey in November 2021 to gather any additional comments on the revised checklists. In line with the updated PRISMA 2020 checklist (and other reporting guidelines, such as TRIPOD, STARD, and CONSORT), a separate checklist for abstracts was also produced and participants were asked for their feedback on this too. The survey was open for two weeks and a reminder was sent. Of the 43 individuals invited, 30 participated in the second survey. The feedback obtained from the second survey was used by the executive committee members in December 2021 to further refine and finalise TRIPOD-SRMA and the accompanying checklist for abstracts. The final checklist was sent around to all members of the executive committee for final approval. The surveys and a summary of the results are provided in appendix 1.

## TRIPOD-SRMA checklist

The TRIPOD-SRMA checklist consists of 26 items (including a total of 34 components) within six sections (table 1): title, abstract, introduction, methods, results, discussion, and other information. Many items in each section remain the same as PRISMA 2020; even though PRISMA 2020 focuses on reviews of studies evaluating intervention effects, several steps are the same when undertaking a systematic review of prediction model studies. Items 1, 2, 4, 5, 10-12, and 15-18 are the most tailored or are included to focus on prediction model reviews specifically. Other PRISMA 2020 items also had a minor change to emphasise the focus of TRIPOD-SRMA on prediction models. For example, item 13 states: “Describe any methods used to assess certainty (or confidence) in the body of evidence for a prediction model.” Another change from PRISMA 2020 is the reference to model performance measures. For example, item 12a includes: “Describe any methods for synthesising estimates of performance measures for each model.” Also, for some items, we explicitly mention the need to report information or results separately for each prediction model of interest; for example, item 18b says: “For each model, present results of all investigations of possible causes of heterogeneity in model performance.” A printable checklist for completion is provided in appendix 2. Table 2 shows the TRIPOD-SRMA checklist for abstracts.

**Table 1:** TRIPOD-SRMA checklist for reporting systematic reviews of prediction model studies

Section and topic	Item No	Checklist item
**Title**		
Title	1	Identify the report as a systematic review or meta-analysis (or both) of diagnostic or prognostic model studies. Specify the target population and outcome(s) predicted as relevant to the review question.
**Abstract**		
Abstract	2	See the TRIPOD-SRMA checklist for abstracts*
**Introduction**		
Rationale	3	Describe the rationale for the review in the context of existing knowledge.
Objectives	4	Provide an explicit statement of the objective(s) being addressed with reference to: target population, index and comparator models (as relevant), outcome(s), time (prediction horizon and intended moment of using the model), and setting.
**Methods**		
Study eligibility criteria	5	Specify study characteristics used as eligibility criteria, including any prediction models of specific interest, and whether development or validation studies (or both) were eligible.
Information sources	6	Specify all databases, registers, websites, organisations, reference lists, and other sources searched or consulted to identify studies. Specify the date when each source was last searched or consulted.
Search strategy	7	Present the full search strategies for all databases, registers, and websites, including any filters and limits used.
Study selection process	8	Specify the methods used to decide whether a study met the inclusion criteria of the review, including how many reviewers screened each record and each report retrieved, whether they worked independently, and if applicable, details of automation tools used in the process.
Data collection process	9	Specify the methods used to collect data from study reports, including how many reviewers collected data from each report, whether they worked independently, any processes for obtaining or confirming data from study investigators, and if applicable, details of automation tools used in the process.
Data Items	10a	List and define all items for which data were sought from each study.
	10b	State the model performance measures that were sought (eg, measures of calibration, discrimination, overall model fit, clinical utility).
	10c	Describe how any desired but unreported data items (items 10a, 10b) were handled (eg, contacted authors, calculated from other reported information).
Risk of bias and applicability assessment	11	Specify the methods used to assess risk of bias in the included studies and their applicability to the review question. This should be done separately for each model development and validation. Include details of any tool(s) used, how many reviewers assessed each study and whether they worked independently.
Synthesis methods	12a	Describe any methods for synthesising estimates of performance measures for each model. If meta-analysis was carried out, describe the methods used, including any transformations of data before pooling, how any heterogeneity in model performance was quantified and handled, and software package(s) used.
	12b	Describe any methods used to explore possible causes of heterogeneity in model performance (eg, subgroup analysis, meta-regression), including whether or not they were planned.
	12c	Describe any sensitivity analyses conducted to assess robustness of the synthesised results.
Certainty assessment	13	Describe any methods used to assess certainty (or confidence) in the body of evidence for a prediction model.
**Results**		
Study selection	14	Describe the results of the search and selection process, from the number of records identified in the search to the number of studies and models included in the review, ideally using a flow diagram.
Study and model characteristics	15	Present study characteristics and model details extracted (as per item 10a), and cite the study reports.
Risk of bias and applicability	16	Present results of risk of bias and applicability assessment. This should be done separately for each model development and validation in each included study.
Results of model performance in individual studies	17	Present performance estimates and confidence intervals for each model and all evaluations, including whether they relate to the internal or external validation performance. If internal, give details of the method.
Results of syntheses	18a	Present the results of any synthesis of model performance, together with details of which study estimates contributed. If meta-analysis was carried out, then for each model and performance measure, present summary results, confidence/credible intervals, and measures of heterogeneity. Forest plots may be useful.
	18b	For each model, present results of all investigations of possible causes of heterogeneity in model performance.
	18c	Present results of all sensitivity analyses conducted to assess the robustness of the synthesised results.
Certainty of evidence	19	Present any assessments of certainty (or confidence) in the body of evidence for each prediction model of interest.
**Discussion**		
Summary of evidence	20	Summarise the main findings including the strengths and limitations of the evidence.
Limitations	21	Discuss the strengths and limitations of the review process.
Implications	22	Discuss implications of the results in the context of other evidence and for practice, policy, and future research.
**Other information**		
Registration and protocol	23a	Provide registration information for the review, including register name and registration number, or state that the review was not registered.
	23b	Indicate where the review protocol can be accessed, or state that a protocol was not prepared.
	23c	Describe and explain any amendments to information provided at registration or in the protocol.
Support	24	Describe sources of financial or non-financial support for the review, and the role of the funders or sponsors in the review.
Competing interests	25	Declare any competing interests of review authors.
Availability of data, code, and other materials	26	Report which of the following are publicly available and where they can be found: template data collection forms; data extracted from included studies; data used for all analyses; analytic code; any other materials used in the review.

TRIPOD-SRMA=transparent reporting of multivariable prediction models for individual prognosis or diagnosis tailored for systematic reviews and meta-analyses.

* See table 2.

**Table 2:** TRIPOD-SRMA checklist for abstracts

Section and topic	Item No	Checklist item
**Title**		
Title	1	Identify the report as a systematic review or meta-analysis (or both) of diagnostic or prognostic model studies. Specify the target population and outcome(s) predicted as relevant to the review question.
**Background**		
Objectives	2	Provide an explicit statement of the main objective(s) being addressed with reference to: target population, index and comparator models (as relevant), outcome(s), time (prediction horizon and intended moment of using the model), and setting.
**Methods**		
Study eligibility criteria	3	Specify study characteristics used as eligibility criteria, including any prediction models of specific interest, and whether development or validation studies (or both) were eligible.
Information sources	4	Specify the information sources (eg, databases, registers) used to identify studies and the date when each was last searched.
Risk of bias and applicability	5	Specify the methods used to assess risk of bias and applicability in the included studies.
Synthesis methods	6	Specify the methods used to synthesise performance measures for each model of interest.
**Results**		
Included studies	7	Give the total number of included studies and models, and summarise relevant study characteristics and model details.
Results of syntheses	8	Present results for each of the main models of interest. If meta-analysis was used to synthesise study estimates of model performance, report the summary result and confidence/credible interval for each performance measure, together with the number of study estimates contributing.
**Discussion**		
Limitations of evidence	9	Provide a brief summary of the limitations of the evidence included in the review.
Interpretation	10	Provide a general interpretation of the results and important implications for research and practice.
**Other**		
Funding	11	Specify the primary source of funding for the review.
Registration	12	Provide the register name and registration number.

## How to use TRIPOD-SRMA

TRIPOD-SRMA is a reporting guideline and thus is not intended to guide how to undertake prediction model reviews; other guidance is available for conduct.^2^ ^18^ ^25^ However, we recommend that reviewers become familiar with the 26 items at the onset of their prediction model review project, because the items can help to provide a broad overview of the key steps and components that such a review involves, and could ultimately help ensure TRIPOD-SRMA can be adhered to when reporting. To aid uptake and understanding, we are preparing an explanation and elaboration document to provide more intricate details and examples for each item, to help reviewers, editors, and readers who require further information or clarity about specific items. We also encourage users to make use of the PRISMA 2020 explanation and elaboration document, which contains an abundance of useful information and examples for systematic reviews in general. Furthermore, the TRIPOD explanation and elaboration document (www.tripod-statement.org) contains a vast amount of information about primary studies of prediction models,^28^ from which many reviewers would benefit from its guidance.

When submitting a prediction model review for publication, we recommend including a form that confirms that each TRIPOD-SRMA item has been adhered to and the location (eg, corresponding page number and subsection heading) where it is contained. To support researchers, a template for TRIPOD-SRMA is provided in appendix 2 and is also available to download and complete from www.tripod-statement.org . If journals impose a word count constraint that makes it difficult to adhere to TRIPOD-SRMA within the main article itself, then the extra information should be provided as supplementary materials or publicly accessible documents, for example. We welcome and encourage translation of TRIPOD-SRMA into different languages, as long as all the authors of the original publication are included in the process and any resulting publication (see www.tripod-statement.org for details on translation).

## Conclusion

The TRIPOD-SRMA checklist and the associated TRIPOD-SRMA checklist for abstracts provide the first reporting guideline for systematic reviews and meta-analyses of prediction model studies. We encourage authors to use TRIPOD-SRMA when writing and publishing such reviews, and we encourage journals and editors to enforce adherence to TRIPOD-SRMA.

## Web extra

Extra material supplied by authors

Web appendix 1: Delphi surveys and results

Web appendix 2: TRIPOD-SRMA Checklist

## Notes

Contributors: The project was initiated by the TRIPOD executive committee (GSC, KGMM, JBR, and RDR), and led throughout by KIES. KIES produced the first draft of the checklist, in consultation with BL. Feedback was provided on the draft checklist by JAAD, PD, TPAD, LH, JBR, GSC, KGMM, and RDR, and the checklist was then revised by KIES. KIES initiated and managed the (modified) Delphi process, including obtaining ethics approval, designing the survey, and summarising feedback and comments received. KIES, BL, and JAAD updated the checklist based on the feedback from the Delphi panel and recommendations from all authors. KIES and RDR wrote the first draft of the article, with important contributions and revisions from all other authors (BL, JAAD, PD, TPAD, LH, JBR, GSC, and KGMM) at all stages. All authors approved the final version submitted. KIES is the guarantor. The corresponding author attests that all listed authors meet authorship criteria and that no others meeting the criteria have been omitted.

Dissemination to participants and related patient and public communities: We plan to disseminate the TRIPOD-SRMA checklist to the EQUATOR Network, to the Cochrane Prognosis Methods Group, via social media, and via various training courses on systematic reviews of prognosis and prediction studies. We also provide templates of the main TRIPOD-SRMA checklist and the TRIPOD-SRMA checklist for abstracts to download and complete from www.tripod-statement.org .

Provenance and peer review: Not commissioned; externally peer reviewed.
