# Supplementary material for: Transparent reporting of multivariable prediction models for individual prognosis or diagnosis: checklist for systematic reviews and meta-analyses (TRIPOD-SRMA)
Source: BMJ. 2023 May 3;381:e073538. doi: 10.1136/bmj-2022-073538 (PMC10155050; doi:10.1136/bmj-2022-073538)
Supplement: Supplementary file 1 — Web appendix 1: Delphi surveys and results [file snek073538.ww1.pdf]

Appendix 1: Delphi surveys and results

TRIPOD-SRMA Delphi survey

Development of a reporting guideline for systematic reviews and meta-analyses of prediction model studies (TRIPOD-SRMA)

Participant details

To ensure this guideline is suitable for a variety of systematic reviews relating to prediction models, it would be helpful to know a little about your experience.

Please let us know which of the following you have experience with: *

Check all that apply
Please select at least one answer
Please choose **all** that apply:

- Developing and validating prognostic prediction models using primary studies
- Developing and validating diagnostic prediction models using primary studies
- Systematic reviews (and meta-analyses) of prognostic model studies
- Systematic reviews (and meta-analyses) of diagnostic model studies
- Other: [ ]

Title and Abstract

Instructions:

Checklist items are listed below and you are asked to indicate the **extent to which you agree that each item should be included** in the checklist.

Please indicate whether you:

(1) **Strongly agree** that the item should be included in the checklist,

(2) **Agree** that the item should be included in the checklist,

(3) **Neither agree nor disagree** that the item should be included in the checklist,

(4) **Disagree** with the item being included in the checklist, or

(5) **Strongly disagree** with the item being included in the checklist.

There is space provided next to each question for (optional) comments on your decision.

The following items relate to the Title and Abstract.

Item 1: Title

Identify the report as a systematic review or meta-analysis (or both) of diagnostic or prognostic model studies. Specify the target population and outcome(s) predicted.

To what extent do you agree that this item should be included in the checklist?

Choose one of the following answers
Please choose **only one** of the following:

- Definitely Agree
- Mostly Agree
- Neither Agree nor Disagree
- Mostly Disagree
- Definitely Disagree

Make a comment on your choice here:

Item 2: Abstract

Provide a structured summary including, as applicable: background, objectives, data sources, study eligibility, target population, setting, outcome(s), study appraisal and synthesis methods, results, limitations, conclusions and implications of key findings, systematic review registration number.

To what extent do you agree that this item should be included in the checklist?

Choose one of the following answers
Please choose **only one** of the following:

- Definitely Agree
- Mostly Agree
- Neither Agree nor Disagree
- Mostly Disagree
- Definitely Disagree

Make a comment on your choice here:

Introduction

The following items relate to the Introduction.

Item 3: Rationale

Describe the rationale for the review in the context of what is already known.

To what extent do you agree that this item should be included in the checklist?

Choose one of the following answers
Please choose **only one** of the following:

- Definitely Agree
- Mostly Agree
- Neither Agree nor Disagree
- Mostly Disagree
- Definitely Disagree

Make a comment on your choice here:

Item 4: Objectives

Provide an explicit statement of questions being addressed with reference to their key elements according to PICOTS: target population, index model, comparator model, outcome(s), time (prediction horizon and intended moment of using the model), and setting.

To what extent do you agree that this item should be included in the checklist?

Choose one of the following answers
Please choose **only one** of the following:

- Definitely Agree
- Mostly Agree
- Neither Agree nor Disagree
- Mostly Disagree
- Definitely Disagree

Make a comment on your choice here:

Methods

The following items relate to the Methods.

Item 5: Eligibility criteria

Specify study characteristics (e.g. in relation to PICOTS), report characteristics (e.g. years considered, language, publication status) and prediction model specific aspects (e.g. specific predictor(s), outcome(s), whether development and validation studies are eligible or only validation studies), used as criteria for eligibility.

To what extent do you agree that this item should be included in the checklist?

Choose one of the following answers
Please choose **only one** of the following:

- Definitely Agree
- Mostly Agree
- Neither Agree nor Disagree
- Mostly Disagree
- Definitely Disagree

Make a comment on your choice here:

Item 6: Information sources

Specify all databases, registers, websites, organisations, reference lists and other sources searched or consulted to identify studies. Specify the date when each source was last searched or consulted.

To what extent do you agree that this item should be included in the checklist?

Choose one of the following answers
Please choose **only one** of the following:

- Definitely Agree
- Mostly Agree
- Neither Agree nor Disagree
- Mostly Disagree
- Definitely Disagree

Make a comment on your choice here:

Item 7: Search strategy

Present the full search strategies for all databases, registers and websites, including any filters and limits used.

To what extent do you agree that this item should be included in the checklist?

Choose one of the following answers
Please choose **only one** of the following:

- Definitely Agree
- Mostly Agree
- Neither Agree nor Disagree
- Mostly Disagree
- Definitely Disagree

Make a comment on your choice here:

Item 8: Selection process

Specify the methods used to decide whether a study met the inclusion criteria of the review, including how many reviewers screened each record and each report retrieved, whether they worked independently, and if applicable, details of automation tools used in the process.

To what extent do you agree that this item should be included in the checklist?

Choose one of the following answers
Please choose **only one** of the following:

- Definitely Agree
- Mostly Agree
- Neither Agree nor Disagree
- Mostly Disagree
- Definitely Disagree

Make a comment on your choice here:

Item 9: Data collection process

Specify the methods used to collect data from reports, including how many reviewers collected data from each report, whether they worked independently, any processes for obtaining or confirming data from study investigators, and if applicable, details of automation tools used in the process.

To what extent do you agree that this item should be included in the checklist?

Choose one of the following answers
Please choose **only one** of the following:

- Definitely Agree
- Mostly Agree
- Neither Agree nor Disagree
- Mostly Disagree
- Definitely Disagree

Make a comment on your choice here:

Item 10: Data items

List and define all variables for which data were sought from each study (e.g. PICOTS, study dates, follow-up duration, country, funding sources, conflicts of interest).

To what extent do you agree that this item should be included in the checklist?

Choose one of the following answers
Please choose **only one** of the following:

- Definitely Agree
- Mostly Agree
- Neither Agree nor Disagree
- Mostly Disagree

- Definitely Disagree

Make a comment on your choice here:

Item 11: Summary measures

Depending on the type of review, state the principal information to be extracted (e.g. model equations, measures of internal or external validation such as the C-statistic, calibration slope, calibration-in-the-large, net benefit).

To what extent do you agree that this item should be included in the checklist?

Choose one of the following answers
Please choose **only one** of the following:

- Definitely Agree
- Mostly Agree
- Neither Agree nor Disagree
- Mostly Disagree
- Definitely Disagree

Make a comment on your choice here:

Item 12: Data transformation

If done, describe any transformations or calculations from the raw data extracted (e.g. scale conversion of C-statistic prior to pooling across studies).

To what extent do you agree that this item should be included in the Choose one of the following answers
Please choose **only one** of the following:

- Definitely Agree
- Mostly Agree
- Neither Agree nor Disagree
- Mostly Disagree
- Definitely Disagree

Make a comment on your choice here:

Item 13: Dealing with unreported information

If applicable, describe how required but unreported information was dealt with (e.g. estimates or confidence intervals of performance measures that were not reported in some validation studies).

To what extent do you agree that this item should be included in the checklist?

Choose one of the following answers
Please choose **only one** of the following:

- Definitely Agree
- Mostly Agree
- Neither Agree nor Disagree
- Mostly Disagree
- Definitely Disagree

Make a comment on your choice here:

Item 14: Risk of bias assessments

Describe how potential sources of bias were assessed (e.g. using PROBAST) for each model reviewed, and how this information was used in the review.

To what extent do you agree that this item should be included in the checklist?

Choose one of the following answers
Please choose **only one** of the following:

- Definitely Agree
- Mostly Agree
- Neither Agree nor Disagree
- Mostly Disagree
- Definitely Disagree

Make a comment on your choice here:

Item 15: Applicability

If relevant, describe how applicability of the models was evaluated with regard to the review question and PICOTS (e.g. using PROBAST).

To what extent do you agree that this item should be included in the checklist?

Choose one of the following answers
Please choose **only one** of the following:

- Definitely Agree
- Mostly Agree
- Neither Agree nor Disagree
- Mostly Disagree
- Definitely Disagree

Make a comment on your choice here:

Item 16: Investigation of heterogeneity

Describe how sources of heterogeneity (e.g. case-mix or setting) between studies were investigated, including if applicable, differences between development and validation studies.

To what extent do you agree that this item should be included in the Choose one of the following answers
Please choose **only one** of the following:

- Definitely Agree
- Mostly Agree
- Neither Agree nor Disagree
- Mostly Disagree
- Definitely Disagree

Make a comment on your choice here:

Item 17: Synthesis of results

If meta-analysis was carried out, describe the methods for pooling performance measures for each model, including how any heterogeneity in model performance was handled (e.g. using random effects) and quantified (e.g. tau-squared, prediction intervals).

To what extent do you agree that this item should be included in the checklist?

Choose one of the following answers
Please choose **only one** of the following:

- Definitely Agree
- Mostly Agree
- Neither Agree nor Disagree
- Mostly Disagree
- Definitely Disagree

Make a comment on your choice here:

Item 18: Certainty assessment

If relevant, describe any methods used to assess certainty (or confidence) in the body of evidence for a prediction model (e.g. using adaptations of GRADE).

To what extent do you agree that this item should be included in the checklist?

Choose one of the following answers
Please choose **only one** of the following:

- Definitely Agree
- Mostly Agree
- Neither Agree nor Disagree
- Mostly Disagree
- Definitely Disagree

Make a comment on your choice here:

Item 19: Additional analyses

Describe any planned subgroup or sensitivity analyses (e.g. pooling a model's predictive performance measures according to settings or risk of bias assessment) or meta-regression.

To what extent do you agree that this item should be included in the checklist?

Choose one of the following answers
Please choose **only one** of the following:

- Definitely Agree
- Mostly Agree
- Neither Agree nor Disagree
- Mostly Disagree
- Definitely Disagree

Make a comment on your choice here:

Results

The following items relate to the Results.

Item 20: Study selection

Describe the results of the search and selection process, from the number of records identified in the search to the number of studies included in the review, ideally using a flow diagram. Also provide the number of models and the number and type of validation in the included studies.

To what extent do you agree that this item should be included in the Choose one of the following answers
Please choose **only one** of the following:

- Definitely Agree
- Mostly Agree
- Neither Agree nor Disagree
- Mostly Disagree
- Definitely Disagree

Make a comment on your choice here:

Item 21: Study characteristics

For each model or study, present characteristics for which data were extracted (e.g. design, sample size, prediction horizon, key study dates, number of participants with each outcome, predictors, treatments received, model equation, performance statistics, whether it was development only, development and internal validation, or external validation, as relevant) and provide citations.

To what extent do you agree that this item should be included in the checklist?

Choose one of the following answers
Please choose **only one** of the following:

- Definitely Agree
- Mostly Agree
- Neither Agree nor Disagree
- Mostly Disagree
- Definitely Disagree

Make a comment on your choice here:

Choose one of the following answers
Please choose **only one** of the following:

- Definitely Agree
- Mostly Agree
- Neither Agree nor Disagree
- Mostly Disagree
- Definitely Disagree

Make a comment on your choice here:

Item 23: Applicability

Report the results from any applicability assessment (e.g. PROBAST) separately for each included model and all validations in each study.

To what extent do you agree that this item should be included in the checklist?

Choose one of the following answers
Please choose **only one** of the following:

- Definitely Agree
- Mostly Agree
- Neither Agree nor Disagree
- Mostly Disagree
- Definitely Disagree

Make a comment on your choice here:

Item 24: Results of individual studies

Present for each model development study: model equation (or link to source code, website, app etc.), estimates (and confidence intervals) of the model's performance measures from apparent or internal validation. Present for each model validation study, estimates of performance measures (and confidence intervals). Note any changes from the original development study.

To what extent do you agree that this item should be included in the Choose one of the following answers
Please choose **only one** of the following:

- Definitely Agree
- Mostly Agree
- Neither Agree nor Disagree
- Mostly Disagree
- Definitely Disagree

Make a comment on your choice here:

Item 25: Synthesis of results

Present results of each meta-analysis (if done), including summary estimates, confidence intervals and measures of heterogeneity in the model's performance measures. Forest plots may be useful.

To what extent do you agree that this item should be included in the checklist?

Choose one of the following answers
Please choose **only one** of the following:

- Definitely Agree
- Mostly Agree
- Neither Agree nor Disagree
- Mostly Disagree
- Definitely Disagree

Make a comment on your choice here:

Item 26: Additional analyses

Report results from any subgroup, sensitivity or meta-regression analyses (see Item 19).

To what extent do you agree that this item should be included in the checklist?

Choose one of the following answers
Please choose **only one** of the following:

- Definitely Agree
- Mostly Agree
- Neither Agree nor Disagree
- Mostly Disagree
- Definitely Disagree

Make a comment on your choice here:

Discussion

The following items relate to the Discussion.

Item 27: Summary of evidence

Summarise the main findings (for each model if relevant) including the strength of evidence (based on external validation performance) and heterogeneity (e.g. in performance across studies).

To what extent do you agree that this item should be included in the checklist?

Choose one of the following answers
Please choose **only one** of the following:

- Definitely Agree
- Mostly Agree
- Neither Agree nor Disagree
- Mostly Disagree
- Definitely Disagree

Make a comment on your choice here:

Item 28: Limitations

Discuss the strengths and limitations at study and model level (e.g. risk of bias) and at review level (e.g. incomplete retrieval of identified research, reporting bias).

To what extent do you agree that this item should be included in the Choose one of the following answers
Please choose **only one** of the following:

- Definitely Agree
- Mostly Agree
- Neither Agree nor Disagree
- Mostly Disagree
- Definitely Disagree

Make a comment on your choice here:

Item 29: Implications

Discuss the findings in the context of other evidence and in the context of the objectives. Consider the relevance to key groups (e.g. healthcare providers, users and policy makers). Discuss any implications/suggestions for potential clinical use and future research.

To what extent do you agree that this item should be included in the checklist?

Choose one of the following answers
Please choose **only one** of the following:

- Definitely Agree
- Mostly Agree
- Neither Agree nor Disagree
- Mostly Disagree
- Definitely Disagree

Make a comment on your choice here:

Item 30: Conclusions

Provide a brief summary of key findings, implications and future research.

To what extent do you agree that this item should be included in the checklist?

Choose one of the following answers
Please choose **only one** of the following:

- Definitely Agree
- Mostly Agree
- Neither Agree nor Disagree
- Mostly Disagree
- Definitely Disagree

Make a comment on your choice here:

Other Information

The following items relate to Other Information.

Item 31: Registration

Provide registration information for the review, including register name and registration number, or state that the review was not registered.

To what extent do you agree that this item should be included in the checklist?

Choose one of the following answers
Please choose **only one** of the following:

- Definitely Agree
- Mostly Agree
- Neither Agree nor Disagree
- Mostly Disagree
- Definitely Disagree

Make a comment on your choice here:

Item 32: Protocol

Indicate where the review protocol can be accessed, or state that a protocol was not prepared.

To what extent do you agree that this item should be included in the checklist?

Choose one of the following answers
Please choose **only one** of the following:

- Definitely Agree
- Mostly Agree
- Neither Agree nor Disagree
- Mostly Disagree
- Definitely Disagree

Make a comment on your choice here:

Item 33: Support and COI

Describe sources of financial or non-financial support for the review (e.g. supply of data), and the role of the funders or sponsors in the review. Declare any potential conflicts of interest for the review authors.

To what extent do you agree that this item should be included in the checklist?

Choose one of the following answers
Please choose **only one** of the following:

- Definitely Agree
- Mostly Agree
- Neither Agree nor Disagree
- Mostly Disagree
- Definitely Disagree

Make a comment on your choice here:

Item 34: Availability of data, code, and other materials

Report which of the following are publicly available and where they can be found: template data collection forms; data extracted from included studies; data used for all analyses; analytic code; any other materials used in the review.

To what extent do you agree that this item should be included in the checklist?

Choose one of the following answers
Please choose **only one** of the following:

- Definitely Agree
- Mostly Agree
- Neither Agree nor Disagree
- Mostly Disagree
- Definitely Disagree

Make a comment on your choice here:

Comments

Are there any items not currently included in TRIPOD-SRMA that you think should be included?

Please write your answer here:

Do you have any other comments or suggestions for TRIPOD-SRMA?

Please write your answer here:

Acknowledgement

Finally, do you consent to be acknowledged by name for your contribution in any resulting academic publications? *

Choose one of the following answers
Please choose **only one** of the following:

- Yes
- No

We appreciate your participation in the Delphi survey for TRIPOD-SRMA. Responses to this survey will be discussed within the TRIPOD group and will inform which items are included in the checklist for the next Delphi survey. All comments will be carefully considered. Thank you for your time.

Reporting guidelines for systematic reviews and meta-analyses of prediction model studies (TRIPOD-SRMA)

Delphi survey results – Round 1

Thank you for participating in Round 1 of our Delphi study to help develop a reporting guideline for systematic reviews and meta-analyses of prediction model studies. Your responses and comments have been very helpful to us as we revise the draft checklist and prepare the accompanying guidance documents.

We are pleased to say that the vast majority of Delphi responders agreed with the inclusion of all items in the draft checklist. However, we have now made modifications to both the items included/excluded and the wording of items, based on Delphi participant feedback.

We are pleased to share a summary of the results from the first Delphi survey.

Out of 86 individuals invited to participate in the Delphi survey, 43 (50%) responded. The experience of responders is given in Table 1 below:

Experience in	Number of participants who said "yes"
Developing and validating prognostic prediction models using primary studies	36
Developing and validating diagnostic prediction models using primary studies	19
Systematic review (and meta-analyses) of prognostic model studies	30
Systematic review (and meta-analyses) of diagnostic model studies	22
Other	6 (other types of systematic reviews including overall prognosis or prognostic factor studies, methodology, IPD meta-analysis)

Below is a summary of the Delphi responses to the questions asking participants to state the level of agreement to each item in the checklist (DA=Definitely agree, MA=Mostly Agree, N=Neither agree nor disagree, MD=Mostly disagree, DD=Definitely disagree).

**TRIPOD-SRMA Checklist items**	**DA**	**MA**	**N**	**MD**	**DD**
**Item 1: Title** Identify the report as a systematic review or meta-analysis (or both) of diagnostic or prognostic model studies. Specify the target population and outcome(s) predicted.	36	7	0	0	0
**Item 2: Abstract** Provide a structured summary including, as applicable: background, objectives, data sources, study eligibility, target population, setting, outcome(s), study appraisal and synthesis methods, results, limitations, conclusions and implications of key findings, systematic review registration number.	26	15	2	0	0
**Item 3: Rationale** Describe the rationale for the review in the context of what is already known.	33	8	1	1	0
**Item 4: Objectives** Provide an explicit statement of questions being addressed with reference to their key elements according to PICOTS: target population, index model, comparator model, outcome(s), time (prediction horizon and intended moment of using the model), and setting.	29	11	1	2	0
**Item 5: Eligibility criteria** Specify study characteristics (e.g. in relation to PICOTS), report characteristics (e.g. years considered, language, publication status) and prediction model specific aspects (e.g. specific predictor(s), outcome(s), whether development and validation studies are eligible or only validation studies), used as criteria for eligibility.	31	10	1	1	0
**Item 6: Information sources** Specify all databases, registers, websites, organisations, reference lists and other sources searched or consulted to identify studies. Specify the date when each source was last searched or consulted.	35	5	2	1	0
**Item 7: Search strategy** Present the full search strategies for all databases, registers and websites, including any filters and limits used.	28	12	2	0	1
**Item 8: Selection process** Specify the methods used to decide whether a study met the inclusion criteria of the review, including how many reviewers screened each record and each report retrieved, whether they worked independently, and if applicable, details of automation tools used in the process.	34	7	2	0	0
**Item 9: Data collection process** Specify the methods used to collect data from reports, including how many reviewers collected data from each report, whether they worked independently, any processes for obtaining or confirming data from study investigators, and if applicable, details of automation tools used in the process.	34	8	1	0	0
**Item 10: Data items** List and define all variables for which data were sought from each study (e.g. PICOTS, study dates, follow-up duration, country, funding sources, conflicts of interest).	27	12	3	1	0
**Item 11: Summary measures** Depending on the type of review, state the principal information to be extracted (e.g. model equations, measures of internal or external validation such as the C-statistic, calibration slope, calibration-in-the-large, net benefit).	35	6	1	1	0
**Item 12: Data transformation** If done, describe any transformations or calculations from the raw data extracted (e.g. scale conversion of C-statistic prior to pooling across studies).	29	4	9	1	0
**Item 13: Dealing with unreported information** If applicable, describe how required but unreported information was dealt with (e.g. estimates or confidence intervals of performance measures that were not reported in some validation studies).	27	10	5	1	0

TRIPOD-SRMA Checklist items	DA	MA	N	MD	DD
**Item 14: Risk of bias assessments** Describe how potential sources of bias were assessed (e.g. using PROBAST) for each model reviewed, and how this information was used in the review.	35	5	2	1	0
**Item 15: Applicability** If relevant, describe how applicability of the models was evaluated with regard to the review question and PICOTS (e.g. using PROBAST).	28	10	4	1	0
**Item 16: Investigation of heterogeneity** Describe how sources of heterogeneity (e.g. case-mix or setting) between studies were investigated, including if applicable, differences between development and validation studies.	31	9	1	2	0
**Item 17: Synthesis of results** If meta-analysis was carried out, describe the methods for pooling performance measures for each model, including how any heterogeneity in model performance was handled (e.g. using random effects) and quantified (e.g. tau-squared, prediction intervals).	37	6	0	0	0
**Item 18: Certainty assessment** If relevant, describe any methods used to assess certainty (or confidence) in the body of evidence for a prediction model (e.g. using adaptations of GRADE).	20	14	7	2	0
**Item 19: Additional analyses** Describe any planned subgroup or sensitivity analyses (e.g. pooling a model's predictive performance measures according to settings or risk of bias assessment) or meta-regression.	24	11	7	1	0
**Item 20: Study selection** Describe the results of the search and selection process, from the number of records identified in the search to the number of studies included in the review, ideally using a flow diagram. Also provide the number of models and the number and type of validation in the included studies.	37	5	1	0	0
**Item 21: Study characteristics** For each model or study, present characteristics for which data were extracted (e.g. design, sample size, prediction horizon, key study dates, number of participants with each outcome, predictors, treatments received, model equation, performance statistics, whether it was development only, development and internal validation, or external validation, as relevant) and provide citations.	33	6	3	0	1
**Item 22: Risk of bias assessments** Present assessments of risk of bias separately for each included model and all validations in each study.	29	8	5	1	0
**Item 23: Applicability** Report the results from any applicability assessment (e.g. PROBAST) separately for each included model and all validations in each study.	27	7	6	3	0
**Item 24: Results of individual studies** Present for each model development study: model equation (or link to source code, website, app etc.), estimates (and confidence intervals) of the model's performance measures from apparent or internal validation. Present for each model validation study, estimates of performance measures (and confidence intervals). Note any changes from the original development study.	26	10	5	2	0
**Item 25: Synthesis of results** Present results of each meta-analysis (if done), including summary estimates, confidence intervals and measures of heterogeneity in the model's performance measures. Forest plots may be useful.	36	5	0	2	0
**Item 26: Additional analyses** Report results from any subgroup, sensitivity or meta-regression analyses (see Item 19).	31	8	2	2	0
**Item 27: Summary of evidence** Summarise the main findings (for each model if relevant) including the strength of evidence (based on external validation performance) and heterogeneity (e.g. in performance across studies).	32	8	1	2	0
**Item 28: Limitations** Discuss the strengths and limitations at study and model level (e.g. risk of bias) and at review level (e.g. incomplete retrieval of identified research, reporting bias).	35	6	1	1	0

TRIPOD-SRMA Checklist items	DA	MA	N	MD	DD
**Item 29: Implications** Discuss the findings in the context of other evidence and in the context of the objectives. Consider the relevance to key groups (e.g. healthcare providers, users and policy makers). Discuss any implications/suggestions for potential clinical use and future research.	31	7	4	1	0
**Item 30: Conclusions** Provide a brief summary of key findings, implications and future research.	34	3	4	1	1
**Item 31: Registration** Provide registration information for the review, including register name and registration number, or state that the review was not registered.	34	7	1	1	0
**Item 32: Protocol** Indicate where the review protocol can be accessed, or state that a protocol was not prepared.	32	8	3	0	0
**Item 33: Support and COI** Describe sources of financial or non-financial support for the review (e.g. supply of data), and the role of the funders or sponsors in the review. Declare any potential conflicts of interest for the review authors.	40	3	0	0	0
**Item 34: Availability of data, code, and other materials** Report which of the following are publicly available and where they can be found: template data collection forms; data extracted from included studies; data used for all analyses; analytic code; any other materials used in the review.	28	9	5	1	0

In line with previous Delphi studies conducted by the group, we considered consensus to be achieved if two thirds of participants agreed with the item's inclusion (if participants 'definitely agreed' or 'mostly agreed') or if two thirds disagreed with the item's inclusion (if participants 'definitely disagreed' or 'mostly disagreed'). By this definition, we achieved consensus on the inclusion of every item in the checklist with a minimum agreement of 76% for any individual item.

Although consensus was achieved, we have modified the checklist items and wording based on the comments left by the participants, as we felt these feedback comments were particularly helpful in the development of the checklist.

For instance, we made the following changes following the feedback:

- Abstract: We have now included a separate checklist in line with PRISMA 2020. Feedback was that the item was not specific enough and separate guidance for writing abstracts would be preferable.
- Objectives: We have removed mention of PICOTS from this item as several responses suggested that it wouldn't always be the right structure for all possible reviews of prediction models. We have also amended wording to be clearer that a comparator model is not always relevant.
- Eligibility criteria: We have shortened the wording for this item as suggested by removing examples. The examples of what to include will instead be discussed in the Explanation & Elaboration (E&E) document that will accompany the checklist. This item now also refers to both study and prediction model characteristics that are used as inclusion and exclusion criteria.
  Item 5: Eligibility Criteria
  Specify study and prediction model characteristics used as inclusion and exclusion criteria, including whether development or validation studies (or both) were eligible.
- Data to be extracted: We have amended the numbering of the previous items 10 and 11 to be 10a and 10b as they both refer to information to be extracted. For Item 10a we now refer

to data items rather than variables, based on feedback. Item 10a now also relates to 'study level' information and includes model details, whereas 10b focuses on predictive performance measures for each prediction model of interest rather than more generic 'summary measures' which had previously included some of the model details. This distinction between 'study' and 'model' levels was also suggested in the feedback. We have removed overlap between PICOTS and other examples listed as suggested. Finally, we no longer mention specific statistics for calibration, discrimination etc. as suggested in the feedback.
Item 10a: Data Items
List and define all items for which data were sought from each study (e.g. PICOTS, study dates, sample size, country, model details, funding sources, conflicts of interest).
Item 10b: Performance measures
State the measures of predictive performance that were sought (e.g. measures of overall model fit, calibration, discrimination, clinical utility).

- Data transformation: General feedback on this item was that it should be removed or merged with another item. Therefore, we have removed this item and included it with the item for synthesis methods (see new item 14 below).

- Dealing with unreported information: Feedback on the item was that it contained too much detail and that there was overlap with the earlier item on the data collection process. Therefore, we have simplified the item wording and made it more distinct from the earlier item.
  Item 11: Dealing with unreported information
  Describe how any required but unreported information was handled (e.g. contacted authors, calculated from other reported information).

- Risk of bias and applicability: Feedback suggested we combine the items on risk of bias assessment and applicability and not mention any specific risk of bias tools. Therefore, the two previous items have now been merged into one and PROBAST is no longer mentioned as an example of a risk of bias tool.
  Item 12: Risk of bias and applicability assessment
  Describe methods used for assessing risk of bias in each study (including how this was used in the review) and for assessing applicability to the review question. This should be done separately for each model development and validation.

- Synthesis methods: Feedback suggested that the items on synthesis should be broader to cover scenarios where meta-analysis is not used. This was addressed in both the methods and results items.
  Item 14: Synthesis methods
  Describe if and how performance measures were summarised for each model. If meta-analysis was carried out, describe the methods used, including any transformations of data prior to pooling and how any heterogeneity in model performance was handled.
  Item 22: Synthesis of results
  Present the results of any synthesis of model performance. If meta-analysis was carried out, for each model present summary estimates, confidence intervals and measures of heterogeneity in performance. Forest plots may be useful.

- Additional analyses: Feedback suggested that authors should have to distinguish between planned and unplanned analyses. Therefore, we have amended the wording.

Item 15: Additional analyses
Describe any subgroup, sensitivity or meta-regression analyses, including whether pre-planned.

- Certainty assessment: There were suggestions to not name specific tools, hence we have removed the example suggesting an adaptation of GRADE.
- Results item on study characteristics: We now include both study and model characteristics to match the item in the methods. We have also simplified the text as suggested in the feedback, by removing the examples which will be discussed in the E&E.
  Item 18: Study and model characteristics
  Present study characteristics and model details extracted (as per Item 10a), including citations.
- Results of individual studies: Feedback on this item was that it was too long, and that model equations weren't always available (e.g. some machine learning algorithms, nomograms etc.). Model details should now be reported as part of a separate item and based on the changes to the items in the methods, this item is now focused on model performance.
  Item 20: Results of model performance
  Present performance estimates and confidence intervals for each model and all evaluations, including whether they relate to the apparent, internal or external performance.
- Additional items: We have now added two new items in the results relating to heterogeneity investigation and certainty of evidence. Although items were included in the methods for these, feedback from participants highlighted that the corresponding items in the results were missing.
- Discussion: Based on the feedback we received, we have modified the items in the discussion to summarise the findings, strengths and limitations of the evidence, and then have a separate item relating to the limitations of the review. This is also in line with PRISMA 2020.
- Conclusion: We have removed the item for conclusions at the end of the discussion, as feedback suggested this was not required in a reporting checklist.

Again, we thank all the Delphi participants for their vital feedback, and we invite you to take part in the second and final Delphi round for TRIPOD-SRMA, in which you will have further opportunity to comment on the revised checklist items.

TRIPOD-SRMA

Development of a reporting guideline for systematic reviews and meta-analyses of prediction model studies (TRIPOD-SRMA)

Second Delphi round

Instructions:

Checklist items are listed below and **you are asked to provide any additional feedback you have for each item.** Please note that in this Delphi round, we are not asking you to state whether you think the items should/should not be included.

Title and Abstract

The following items relate to the Title and Abstract.

Item 1: Title

Identify the report as a systematic review or meta-analysis (or both) of diagnostic or prognostic model studies. Specify the target population and outcome(s) predicted as relevant to the review question.

(modified item from another checklist e.g. PRISMA or TRIPOD-Cluster)

Do you have any comments about this item?

Please write your answer here:

Item 2: Abstract

See the TRIPOD-SRMA Checklist for Abstracts.

Checklist for Abstracts

Section and topic	*Item No*	*Checklist item*
Title		

Title	*1*	*Identify the report as a systematic review or meta-analysis (or both) of diagnostic or prognostic model studies. Specify the target population and outcome(s) predicted as relevant to the review question.*
Background		
Objectives	*2*	*Provide an explicit statement of the main objective(s) the review addresses with reference to: target population, index and comparator models (as relevant), outcome(s), time (prediction horizon and intended moment of using the model) and setting.*
Methods		
Eligibility criteria	*3*	*Specify the study and model inclusion criteria for the review.*
Information sources	*4*	*Specify the information sources (e.g. databases, registers) used to identify studies and the date when each was last searched.*
Risk of bias and applicability	*5*	*Specify the methods used to assess risk of bias and applicability in the included studies.*
Synthesis methods	*6*	*Specify the methods used to present and synthesise performance measures for each model of interest.*
Results		
Included studies	*7*	*Give the total number of included studies and models, and summarise relevant study characteristics and model details.*
Synthesis of results	*8*	*Present results for the main models of interest. If meta-analysis was used to synthesise study estimates of model performance, report the summary result and confidence/credible interval for each performance measure.*
Discussion		
Limitations of evidence	*9*	*Provide a brief summary of the limitations of the evidence included in the review.*
Interpretation	*10*	*Provide a general interpretation of the results and important implications for research and practice.*
Other		
Funding	*11*	*Specify the primary source of funding for the review.*
Registration	*12*	*Provide the register name and registration number.*

(modified item from another checklist e.g. PRISMA or TRIPOD-Cluster)

Do you have any comments about this item?

Please write your answer here:

Introduction

The following items relate to the Introduction.

Item 3: Rationale

Describe the rationale for the review in the context of existing knowledge.

(Unchanged item from PRISMA 2020)

Do you have any comments about this item?

Please write your answer here:

Item 4: Objectives

Provide an explicit statement of the objective(s) being addressed with reference to: target population, index and comparator models (as relevant), outcome(s), time (prediction horizon and intended moment of using the model), and setting.

(modified item from another checklist e.g. PRISMA or TRIPOD-Cluster)

Do you have any comments about this item?

Please write your answer here:

Methods

The following items relate to the Methods.

Item 5: Eligibility criteria

Specify study and prediction model characteristics used as inclusion and exclusion criteria, including whether development or validation studies (or both) were eligible.

(modified item from another checklist e.g. PRISMA, TRIPOD-Cluster, Cochrane)

Do you have any comments about this item?

Please write your answer here:

Item 6: Information sources

Specify all databases, registers, websites, organisations, reference lists and other sources searched or consulted to identify studies. Specify the date when each source was last searched or consulted.

(Unchanged item from PRISMA 2020)

Do you have any comments about this item?

Please write your answer here:

Item 7: Search strategy

Present the full search strategies for all databases, registers and websites, including any filters and limits used.

(Unchanged item from PRISMA 2020)

Do you have any comments about this item?

Please write your answer here:

Item 8: Selection process

Specify the methods used to decide whether a study met the inclusion criteria of the review, including how many reviewers screened each

record and each report retrieved, whether they worked independently, and if applicable, details of automation tools used in the process.

(Unchanged item from PRISMA 2020)

Do you have any comments about this item?

Please write your answer here:

Item 9: Data collection process

Specify the methods used to collect data from reports, including how many reviewers collected data from each report, whether they worked independently, any processes for obtaining or confirming data from study investigators, and if applicable, details of automation tools used in the process.

(Unchanged item from PRISMA 2020)

Do you have any comments about this item?

Please write your answer here:

Item 10a: Data items

List and define all items for which data were sought from each study (e.g. PICOTS, study dates, sample size, country, model details, funding sources, conflicts of interest).

(modified item from another checklist e.g. PRISMA, TRIPOD-Cluster, Cochrane)

Do you have any comments about this item?

Please write your answer here:

Item 10b: Performance measures

State the measures of predictive performance that were sought (e.g. measures of overall model fit, calibration, discrimination, clinical utility).

(modified item from another checklist e.g. PRISMA, TRIPOD-Cluster, Cochrane)

Do you have any comments about this item?

Please write your answer here:

Item 11: Dealing with unreported information

Describe how any required but unreported information was handled (e.g. contacted authors, calculated from other reported information).

(New item)

Do you have any comments about this item?

Please write your answer here:

Item 12: Risk of bias and applicability assessment

Describe methods used for assessing risk of bias in each study (including how this was used in the review) and for assessing applicability to the review question. This should be done separately for each model development and validation.

(modified item from another checklist e.g. PRISMA, TRIPOD-Cluster, Cochrane, PRISMA-DTA)

Do you have any comments about this item?

Please write your answer here:

Item 13: Investigation of heterogeneity

Describe if and how sources of heterogeneity between studies were investigated.

(modified item from another checklist e.g. PRISMA, TRIPOD-Cluster, Cochrane)

Do you have any comments about this item?

Please write your answer here:

Item 14: Synthesis methods

Describe if and how performance measures were summarised for each model. If meta-analysis was carried out, describe the methods used, including any transformations of data prior to pooling and how any heterogeneity in model performance was handled.

(modified item from another checklist e.g. PRISMA, TRIPOD-Cluster, Cochrane)

Do you have any comments about this item?

Please write your answer here:

Item 15: Additional analyses

Describe any subgroup, sensitivity or meta-regression analyses, including whether pre-planned.

(modified item from another checklist e.g. PRISMA, TRIPOD-Cluster, Cochrane)

Do you have any comments about this item?

Please write your answer here:

Item 16: Certainty assessment

Describe any methods used to assess certainty (or confidence) in the body of evidence for a prediction model.

(modified item from another checklist e.g. PRISMA, TRIPOD-Cluster, Cochrane)

Do you have any comments about this item?

Please write your answer here:

Results

The following items relate to the Results.

Item 17: Study selection

Describe the results of the search and selection process, from the number of records identified in the search to the number of studies and models included in the review, ideally using a flow diagram.

(modified item from another checklist e.g. PRISMA, TRIPOD-Cluster, Cochrane)

Do you have any comments about this item?

Please write your answer here:

Item 18: Study and model characteristics

Present study characteristics and model details extracted (as per Item 10a), including citations.

(modified item from another checklist e.g. PRISMA, TRIPOD-Cluster, Cochrane)

Do you have any comments about this item?

Please write your answer here:

Item 19: Risk of bias and applicability

Present results of risk of bias and applicability assessment. This should be done separately for each model development and validation.

(modified item from another checklist e.g. PRISMA, TRIPOD-Cluster, Cochrane)

Do you have any comments about this item?

Please write your answer here:

Item 20: Results of model performance

Present performance estimates and confidence intervals for each model and all evaluations, including whether they relate to the apparent, internal or external performance.

(modified item from another checklist e.g. PRISMA, TRIPOD-Cluster, Cochrane)

Do you have any comments about this item?

Please write your answer here:

Item 21: Results of heterogeneity

For each model, present the results of any investigations of heterogeneity.

(modified item from another checklist e.g. PRISMA, TRIPOD-Cluster, Cochrane)

Do you have any comments about this item?

Please write your answer here:

Item 22: Synthesis of results

Present the results of any synthesis of model performance. If meta-analysis was carried out, for each model present summary estimates, confidence intervals and measures of heterogeneity in performance. Forest plots may be useful.

(modified item from another checklist e.g. PRISMA, TRIPOD-Cluster, Cochrane)

Do you have any comments about this item?

Please write your answer here:

Item 23: Results of additional analyses

Report results from any subgroup, sensitivity or meta-regression analyses.

(modified item from another checklist e.g. PRISMA, TRIPOD-Cluster, Cochrane)

Do you have any comments about this item?

Please write your answer here:

Item 24: Certainty of evidence

Present any assessments of certainty (or confidence) in the body of evidence for each prediction model of interest.

(modified item from another checklist e.g. PRISMA, TRIPOD-Cluster, Cochrane)

Do you have any comments about this item?

Please write your answer here:

Discussion

The following items relate to the Discussion.

Item 25: Summary of evidence

Summarise the main findings including the strengths and limitations of the evidence.

(modified item from another checklist e.g. PRISMA, TRIPOD-Cluster, Cochrane)

Do you have any comments about this item?

Please write your answer here:

Item 26: Limitations

Discuss the strengths and limitations of the review process.

(modified item from another checklist e.g. PRISMA, TRIPOD-Cluster, Cochrane)

Do you have any comments about this item?

Please write your answer here:

Item 27: Implications

Discuss implications of the results in the context of other evidence and for practice, policy, and future research.

(modified item from another checklist e.g. PRISMA, TRIPOD-Cluster, Cochrane)

Do you have any comments about this item?

Please write your answer here:

Other Information

The following items relate to Other Information.

Item 28a: Registration

Provide registration information for the review, including register name and registration number, or state that the review was not registered.

(Unchanged item from PRISMA 2020)

Do you have any comments about this item?

Please write your answer here:

Item 28b: Protocol

Indicate where the review protocol can be accessed, or state that a protocol was not prepared.

(Unchanged item from PRISMA 2020)

Do you have any comments about this item?

Please write your answer here:

Item 28c: Protocol amendments

Describe and explain any amendments to information provided at registration or in the protocol.

(Unchanged item from PRISMA 2020)

Do you have any comments about this item?

Please write your answer here:

Item 29a: Support

Describe sources of financial or non-financial support for the review (e.g. supply of data), and the role of the funders or sponsors in the review.

(Unchanged item from PRISMA 2020)

Do you have any comments about this item?

Please write your answer here:

Item 29b: COI

Declare any competing interests of review authors.

(Unchanged item from PRISMA 2020)

Do you have any comments about this item?

Please write your answer here:

Item 30: Availability of data, code, and other materials

Report which of the following are publicly available and where they can be found: template data collection forms; data extracted from included studies; data used for all analyses; analytic code; any other materials used in the review.

(Unchanged item from PRISMA 2020)

Do you have any comments about this item?

Please write your answer here:

Comments

Do you have any other comments or suggestions for TRIPOD-SRMA?

Please write your answer here:

We appreciate your participation in the Delphi survey for TRIPOD-SRMA. Responses to this survey will be discussed within the TRIPOD group and will inform the final checklist and item wording. All comments will be carefully considered. Thank you for your time.
